# Analyzing Gene Expression from Whole Tissue vs. Different Cell Types Reveals the Central Role of Neurons in Predicting Severity of Alzheimer’s Disease

**DOI:** 10.1371/journal.pone.0045879

**Published:** 2012-09-28

**Authors:** Shiri Stempler, Eytan Ruppin

**Affiliations:** 1 The Sackler School of Medicine, Tel Aviv University, Tel Aviv, Israel; 2 The Blavatnik School of Computer Science, Tel Aviv University, Tel Aviv, Israel; McGill University, Canada

## Abstract

Alterations in gene expression resulting from Alzheimer’s disease have received considerable attention in recent years. Although expression has been investigated separately in whole brain tissue, in astrocytes and in neurons, a rigorous comparative study quantifying the relative utility of these sources in predicting the progression of Alzheimer’s disease has been lacking. Here we analyze gene expression from neurons, astrocytes and whole tissues across different brain regions, and compare their ability to predict Alzheimer’s disease progression by building pertaining classification models based on gene expression sets annotated to different biological processes. Remarkably, we find that predictions based on neuronal gene expression are significantly more accurate than those based on astrocyte or whole tissue expression. The findings explicate the central role of neurons, particularly as compared to glial cells, in the pathogenesis of Alzheimer’s disease, and emphasize the importance of measuring gene expression in the most relevant (pathogenically ‘proximal’) single cell types.

## Introduction

Alzheimer’s disease (AD) is the most common cause of dementia and its prevalence is rapidly increasing. AD is characterized by impairment in cognitive processes and its neuropathology is characterized by the intra-cellular neurofibrillary tangles and the extra-cellular β-amyloid (Aβ) plaques [1], [2], [3]. These markers are found mainly in the neocortex and limbic cortex, including the hippocampus and entorhinal cortex. Braak and Braak have proposed a staging scheme, which is based on the density and distribution of neurofibrillary tangles [4]. This staging shows anatomical progression through the brain, from entorhinal (Braak stages I–II), to limbic (Braak stages III–IV), through to isocortical regions (Braak stages V–VI) [5]. At present, there are no treatments that can stop the neurodegenerative process [6].

The brain contains various cell types with astrocytes being the most abundant [7]. The nature of neuron–glia interactions in controlling the function and pathology of our brains remains quite a mystery in neurobiology due to limitations of procedures that allow cell purification [8]. Astrocytes perform many control and regulatory functions and are known to compose a heterogeneous cell population [9]. In the last decade, neurobiologists have shifted their view of astrocytes from supporting cell types to multifunctional housekeeping cells. Much attention has been paid to the role of astrocytes in AD [7] and Aβ peptides have been shown able to activate astrocytes [10], [11]. However, in contrast to neurons, we have limited knowledge about the functional diversity of astrocytes [12].

Changes in gene expression have become a major focus of neurodegenerative disease research [5]. These alterations provide clues about the mechanisms involved in the pathogenesis of diseases and may aid in discovering novel drugs. Hippocampal transcriptional profiling has been the focus of AD studies due to its involvement in memory and spatial navigation, which are being damaged early in the disease [13], [14]. Microarrays of both whole tissues and of isolated neurons have been reported for the hippocampus as well as for other brain regions [15], [16], [17], [18]. In addition, a microarray of cortical astrocytes was recently reported [19].

As reviewed above, the analysis of gene expression changes in AD has already been investigated separately in whole tissue, in astrocytes and in neurons. However, to the best of our knowledge, up until now no one has compared between all these different sources of gene expression and asked which provides the most information about the progression of AD. This question is of paramount interest, since it provides a strong clue to the main culprit of AD, and might help to elucidate the initial events causing AD. Given this data, we could build predictors of AD progression and thereby to measure the association (information content) of gene expression from various tissues and cell-types with the progression of the disease.

A recent study comparing the ability to predict AD progression by the expression of neuronal metabolic genes versus whole tissue metabolic genes in the hippocampus region has pointed to the importance of altered metabolic processes in the neuronal cells [20]. In the current study, we examine this question on a markedly broader scope by generating predictors based on a wider set of metabolic genes, on other Gene Ontology (GO) [21] biological processes, and performing the analysis in a host of different brain regions. Furthermore, astrocyte gene expression is used for the first time for building predictors of AD progression and is compared with neuronal cells expression and whole tissues results. Taken together, this enables us to study the dependence of this fundamental relation between gene expression and AD progression on the specific functional annotations of genes.

## Results

**Figure 1:**
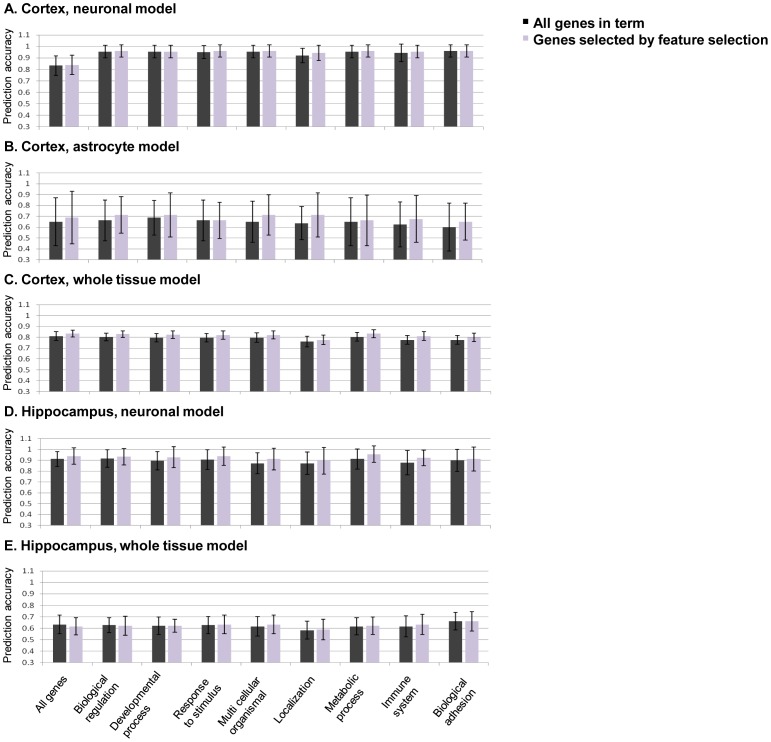
Mean accuracies of predictions of AD severity obtained from various classification models. Each bar represents the mean accuracy of 20 classification models built using cross-validation based on cortex (A) neuronal (control, NDAD and AD samples), (B) astrocytes (early and advanced AD samples) and (C) whole tissue (control and AD samples) and on hippocampus (D) neuronal (control, NDAD and AD samples) and (E) whole tissue (control and AD samples) gene expression data, using all available genes (leftmost columns) or genes from specific biological processes. Standard deviations (SD) are shown as error bars. Two classifiers results are presented for each case: one classifier using all genes annotated to that biological process, and another classifier that imposes an additional feature selection for only top selected genes (see Methods).

In order to investigate the role of different biological processes in AD we have selected several GO terms which are the top categories in the GO process hierarchy [21] (Table 1). To compare the influence of gene expression alterations in the disease in whole tissue to that of specific cell types, we built classification models utilizing different available datasets containing gene expression from two different regions: the cortex and the hippocampus. For each dataset we generated models using several different sets of genes, annotated to different biological processes as mentioned above. The three cortical datasets used of whole tissue, neurons and astrocytes are detailed in Table 2. All gene expression datasets were preprocessed as detailed in the Methods section. Classification models for the disease stages available for each dataset (see Table 2) were generated using the widely used random forest algorithm (Methods). Separate models were produced for different subsets of the data in order to compare the ability of genes involved in particular biological processes (as determined by GO term) to predict the progression of AD. For feature selection, we generated models based on different cutoffs on the number of genes selected (Methods). The prediction accuracy obtained in each analysis was evaluated on unseen test data, and the cutoff obtaining the most accurate results was chosen (Table S1). Accuracies of the 20 cross-validation models based on the chosen cutoff for each tissue sample type and biological process are shown in Figure 1A–C. (A permutation test yielded p-value <0.01 for the neuronal and whole cortex models and p-value of 0.05 for the astrocyte model, see Methods). Additional classification algorithms that are commonly used including support vector machine (SVM) and decision tree were examined as well and similar accuracies were obtained (Table S2). The top AD predictive genes in each of the models are listed in Table S3.

**Table 1:** The list of GO terms used in the current study and the number of genes annotated to each process.

GO term	Number of genes
Biological adhesion	670
Biological regulation	8239
Developmental process	3295
Immune system process	1064
Localization	236
Metabolic process	7803
Multicellular organismal process	3028
Response to stimulus	5551

**Table 2:** Datasets used for generating the classification models.

Reference	Classes in the model	Number of samples	Tissue/cell type	Brain region	Dataset
[25]	Control, AD	364	Whole tissue	Cortex (mostly temporal)	GSE15222
[16], [17]	Control, NDAD*, AD	29	Neurons	Entorhinal cortex	GSE5281
[19]	Early AD, advanced AD	18	Astrocytes	Temporal cortex	GSE29652
[15]	Control, incipient AD, advanced AD	31	Whole tissue	Hippocampus	GSE1297
[16], [17]	Control, NDAD*, AD	29	Neurons	Hippocampus	GSE5281

* NDAD are non-demented individuals with intermediate AD neuropathology.

Interestingly, comparison of the prediction results of the different models revealed high prediction accuracy when relying on entorhinal cortex neuronal gene expression across the different biological processes (Figures 1 and S1, prediction accuracy >0.9). A similar analysis of neuronal dataset from an additional temporal lobe region (middle temporal gyrus, included in GSE5281) has also obtained high prediction accuracies of ∼0.9 (Figure S2). Furthermore, the astrocyte models (prediction accuracy <0.71) yielded lower results than the whole tissue models (prediction accuracy ∼0.8). Neuronal models that classify only between two AD severity classes (control vs. AD or NDAD vs. AD, to be exactly on par with the disease categories available for the whole tissue and astrocyte datasets) performed similarly well to the 3-class neuronal-based models (accuracy >0.9 in both cases), still outperforming other tissue types. Since the whole tissue includes both neuron and astrocyte cell types, its results may represent the averaging of the prediction accuracies obtained via the neurons and via the astrocytes in isolation. This effect of heterogeneity of cells, which confounds the expression profile in whole tissue profiling, was reviewed by [22], raising the potential importance of single-cell gene-expression in medicine. Notably, the prediction accuracies were similar when analyzing different groups of genes which are annotated to different biological processes, both for the models which are based on whole tissue and the models based on a specific cell type.

The analysis above compares the different cell-type sources, all arising from the temporal cortex, but due to the constraints imposed by the existing data, these data come from different subregions within the temporal cortex. To control for potential differences that may arise in classification accuracies due to these sub-regional differences, we have repeated our analysis using data from an additional brain region – the hippocampus – which is also affected severely by AD.

Hippocampal classification models were built using data from whole tissue microarrays [15] and from neuronal microarrays [15]–[16] (regrettably, astrocytic gene expression was not available for the hippocampus) (see Table 2). As before, classification models for the prediction of AD severity stages were generated using sets of genes annotated to a number of biological processes in addition to using all genes in the microarray. Feature selection was performed as detailed above for the cortical datasets and the prediction accuracy results are shown in Figure 1D–E (P-value<0.01 for both neuronal and whole hippocampus models (Methods)). The top AD predictive genes in each of the models are listed in Table S3. Reassuringly, the prediction accuracy of the neuronal models is markedly higher than the whole tissue data models (even to a much larger extent than the difference observed when analyzing the cortical region models). These results are consistent with the dilution effect that was suggested to occur in regional hippocampus microarrays in AD [18]. Taken together, the results highlight the role of gene expression data from neuronal cells in predicting the progression of AD, and show that their expression is markedly more informative regarding the latter than expression of astrocytes or of the tissue as a whole. Furthermore, as the prediction results were very similar when testing models based on different biological process annotations, it appears that many different subsets of genes can be equally predictive of AD.

## Discussion

The human brain is heterogeneously composed of distinct regions and a variety of cell types that can be differentially affected in a disease. In our current study we compared microarray gene expression datasets from neurons, astrocytes and whole tissue microarrays from brains of healthy and AD patients, assessing the ability of various models, applied to different cellular processes, cell types, and brain regions, to predict AD and AD progression. We use expression data from both the cortex and the hippocampus brain regions, which are both known to be involved in the disease.

Our key finding is that neuronal cells possess the information needed to accurately predict AD progression in all the different brain regions studied (Figure S1), and are markedly superior in predicting the disease than either astrocytes or whole tissue. The accuracies of models across the different biological processes in predicting AD based on entorhinal cortex and hippocampus neuronal expression were all higher than 0.9 (Figures 1 and S1). Prediction accuracies based on entorhinal cortex region were slightly higher than those based on the hippocampus genes for the early stage of the disease. This is to be compared with prediction accuracies which were lower than 0.71 for astrocytes models (of the temporal cortex) and accuracies of ∼0.8 and ∼0.6 for whole tissue models based on cortex (mostly temporal cortex) and hippocampus data, respectively, highlighting the key role of neurons in the pathogenesis of AD.

Interestingly, astrocytes provided the lowest predictions of AD progression, even when comparing their results to the whole cortical tissue models. As mentioned earlier, astrocytic cells are heterogeneous. Therefore, although the astrocytes were isolated based on GFAP marker in the reported study, it is possible that within this GFAP^+^ population there is still significant variation. Furthermore, there might be non-GFAP^+^ populations of astrocytes that could yield better predictions of AD progression, but this will not be possible until a better astrocyte marker is identified [19].

When generating models based on whole tissue, the results are likely to represent the overall picture of the tissue, and therefore to resemble an averaging of the different cell types present in the tissue, which may explain the finding that whole tissue data yields accuracies between those of astrocytes and neurons. As mRNA levels are highly variable even within a homogeneous cell population [12], single cell expression analysis may also lead to improved future prediction efforts. Notably, we obtained similar prediction results among different subgroups of genes annotated to different biological processes. This may be due on the one hand to the strong correlations that may exist between the expression of various genes in the cells, and on the other hand due to the large diversity of genes that are affected in a complex disease such as AD (as noted, e.g., by [23]). Interestingly, several of these subgroups of genes contained AD related genes including APP, PSEN1 and PSEN2. Mutations in these genes are known to cause early-onset familial AD, but have unclear roles in late onset AD (the subject of this study) [24]. However, when employing the feature selection method which chooses the top predictive genes in an unbiased way, only APP was selected as top predictive of AD and only in models based on the hippocampus neuronal dataset (Table S3). Nevertheless, other subgroups of genes that did not include APP could predict AD progression with similar accuracy.

In summary, various classification models were generated based on gene expression from whole tissues, neurons and astrocytes from different brain regions, and their prediction accuracies of AD severity levels were compared. This study has revealed that neuronal gene expression is an excellent predictor of AD initiation and progression in all brain regions studied. As astrocyte gene expression provides much lower prediction accuracy, these results strongly suggest, on a genome scale, that the gene expression changes that neurons undergo during AD progression are more profound than those observed in the astrocytes (according to currently available astrocyte microarrays). Furthermore, the inferior results obtained by the whole tissue models from the two brain regions highlight the importance of isolating single cell types for the study of AD pathology and for the prediction of phenotypic changes. With the advancement of methods and markers enabling better isolation of different cells subpopulations, the investigation of their prediction ability should be further studied and is expected to lead to even more accurate predictions. Finally, the methodological lessons learnt from this study are likely to apply to the study of gene expression of other tissues and organs in humans.

## Materials and Methods

### 2.1 Datasets

All microarray data used in this study were obtained from the Gene Expression Omnibus site [25] and are detailed in Table 2.

For the analysis of cortical regions we used 3 different gene expression datasets: (1) for whole cortex analysis we used microarray GSE15222, which contains control and AD cortical samples (mainly temporal cortex) [26]; (2) for the neuronal analysis we used microarrays from laser capture microdissected non-tangle bearing neurons from the entorhinal cortex (located in the temporal lobe) (GSE5281) [16], [17]; and (3) for the analysis of astrocytes we used dataset GSE29652 which contains gene expression data from astrocytes isolated from lateral temporal cortex with different AD severity levels (determined by their Braak stage: I-II, III-IV and V-VI) [19].

For the analysis of the hippocampus region we used two gene expression datasets (Table 2): (1) whole hippocampus tissue samples (GSE1297) [15]; and (2) expression profiles of laser capture microdissected non-tangle bearing hippocampal neurons [16], [17].

Further details on the samples that were used such as post-mortem intervals, age and gender can be found in the original gene expression microarray papers.

Each dataset was filtered several times according to the groups of genes annotated to the different GO slim terms (the top categories in the GO process hierarchy) that were used (Table 1) [21].

### 2.2 Classification Models

The total samples of each dataset were randomly divided to training and test sets, consisting of 2/3 and 1/3 of the samples, respectively. This process was repeated 20 times to obtain 20 random partitions for each dataset in a standard cross-validation procedure. Each of the training and test sets were sampled such that they contained similar ratios of severity classes to the entire dataset. See Table 2 for details on the classes that were used in the different models.

The random forest classification algorithm was used for generating the classification models [27]. The Matlab implementation of random forest was trained on the gene expression data training sets (http://code.google.com/p/randomforest-matlab/). Default parameters were used (500 trees were grown). Model training and performance evaluation were done on distinct subsets of data.

Separate models were generated for different subsets of genes (see Table 1) taken from each of the whole tissue and cell types datasets detailed above, in order to compare the ability of genes involved in particular biological processes (as determined by GO term) to predict the progression of AD.

### 2.3 Feature (Gene) Selection

For feature (gene) selection, the genes with the highest importance values obtained from the models in each of the different random partitions were selected. Different cutoffs of genes with highest importance scores were chosen and the same training data was used to retrain a model using the genes that were above the cutoff point. The cutoff that obtained the highest prediction accuracy for the classification models was chosen to obtain the final list of selected genes in each of the various biological processes and different datasets models (see Table S1). Prediction accuracy is defined as the number of true predictions divided by the number of samples in a test set.

### 2.4. Determining the Statistical Significance of the Models

To assess the significance of the classification models’ prediction accuracy, a permutation test was applied [28]. In each permutation test, the class labels were randomly assigned to each sample, and the entire model discovery process was repeated. For each of the 20 partitions, 100 such permuted data sets were produced and the permutation p-value was computed. This test was repeated for each of the models that were generated in all the different datasets that were used.

## Supporting Information

Figure S1 **Mean accuracies of predictions of Alzheimer’s disease (AD) severity obtained from various feature selection classification models.** Each bar represents the mean accuracy of 20 classification models built using cross-validation based on neuronal (control, NDAD and AD samples) gene expression data from (A) Entorhinal cortex and (B) Hippocampus, using groups of genes annotated to specific biological processes. SD are shown as error bars (see Methods).
(DOC)

Figure S2 **Mean accuracies of predictions of AD severity obtained from various classification models.** Each bar represents the mean accuracy of 20 classification models built using cross-validation based on middle temporal gyrus neuronal (control, NDAD and AD samples) gene expression data, using all available genes (leftmost columns) or genes from specific biological processes. SD are shown as error bars. Two classifiers results are presented for each case: one classifier using all genes annotated to that biological process, and another classifier that imposes an additional feature selection for only top selected genes (see Methods).
(DOC)

Table S1 **Number of genes that were selected for the different feature selection classification models of the various biological processes.**
(DOC)

Table S2 **Mean prediction accuracies obtained by different biological processes classification models by using SVM and decision tree algorithms.**
(DOC)

Table S3 **Lists of top 50 AD predictive genes that were most frequent in the different feature selection models.**
(XLSX)
