# Correction: Analyzing Gene Expression from Whole Tissue vs. Different Cell Types Reveals the Central Role of Neurons in Predicting Severity of Alzheimer’s Disease

**DOI:** 10.1371/annotation/e5e824bf-e57f-4225-bfa0-cc32241a1ff0

**Published:** 2012-10-22

**Authors:** Shiri Stempler, Eytan Ruppin

There were errors in the Funding section. The correction funding information is as follows: S.S. gratefully acknowledges the support of the Josef Sagol Fellowship for brain research at Tel Aviv University. This research was supported by the I-CORE Program of the Planning and Budgeting Committee and The Israel Science Foundation (grant No 41/11). The funders had no role in study design, data collection and analysis, decision to publish, or preparation of the manuscript. 

