# Supplementary material for: Analyzing Gene Expression from Whole Tissue vs. Different Cell Types Reveals the Central Role of Neurons in Predicting Severity of Alzheimer’s Disease
Source: PLoS One. 2012 Sep 28;7(9):e45879. doi: 10.1371/journal.pone.0045879 (PMC3461041; doi:10.1371/journal.pone.0045879)
Supplement: Figure S1 — Mean accuracies of predictions of Alzheimer’s disease (AD) severity obtained from various feature selection classification models. Each bar represents the mean accuracy of 20 classification models built using cross-validation based on neuronal (control, NDAD and AD samples) gene expression data from (A) Entorhinal cortex and (B) Hippocampus, using groups of genes annotated to specific biological processes. SD are shown as error bars (see Methods). (DOC) [file pone.0045879.s001.doc]

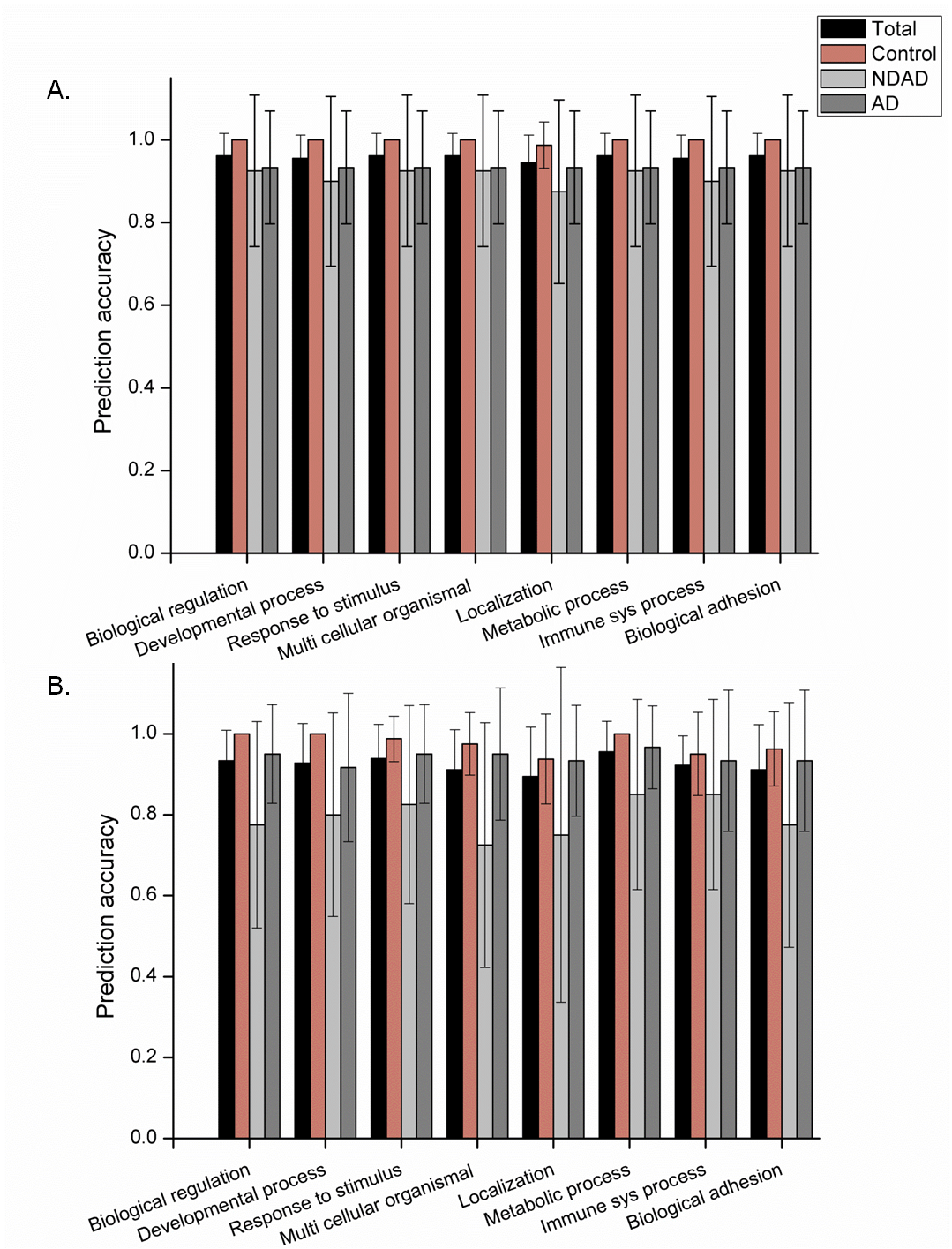


**Figure S1. Mean accuracies of predictions of Alzheimer's disease (AD) severity obtained from various feature selection classification models.** Each bar represents the mean accuracy of 20 classification models built using cross-validation based on neuronal (control, NDAD and AD samples) gene expression data from (A) Entorhinal cortex and (B) Hippocampus, using groups of genes annotated to specific biological processes. Standard deviations (SD) are shown as error bars (see Methods).
