# Supplementary material for: Analyzing Gene Expression from Whole Tissue vs. Different Cell Types Reveals the Central Role of Neurons in Predicting Severity of Alzheimer’s Disease
Source: PLoS One. 2012 Sep 28;7(9):e45879. doi: 10.1371/journal.pone.0045879 (PMC3461041; doi:10.1371/journal.pone.0045879)
Supplement: Figure S2 — Mean accuracies of predictions of AD severity obtained from various classification models. Each bar represents the mean accuracy of 20 classification models built using cross-validation based on middle temporal gyrus neuronal (control, NDAD and AD samples) gene expression data, using all available genes (leftmost columns) or genes from specific biological processes. SD are shown as error bars. Two classifiers results are presented for each case: one classifier using all genes annotated to that biological process, and another classifier that imposes an additional feature selection for only top selected genes (see Methods). (DOC) [file pone.0045879.s002.doc]

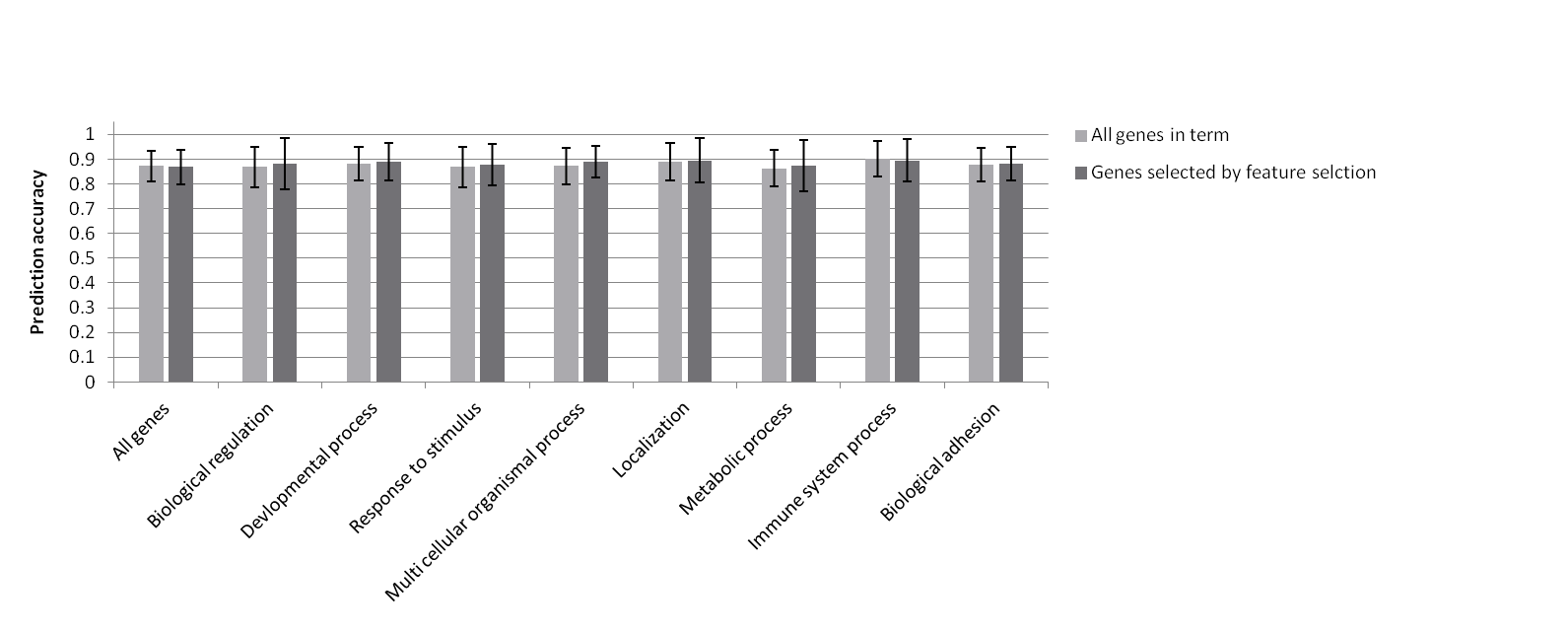


**Figure S2. Mean accuracies of predictions of AD severity obtained from various classification models.** Each bar represents the mean accuracy of 20 classification models built using cross-validation based on middle temporal gyrus neuronal (control, NDAD and AD samples) gene expression data, using all available genes (leftmost columns) or genes from specific biological processes. SD are shown as error bars. Two classifiers results are presented for each case: one classifier using all genes annotated to that biological process, and another classifier that imposes an additional feature selection for only top selected genes (see Methods).
