# Supplementary material for: Analyzing Gene Expression from Whole Tissue vs. Different Cell Types Reveals the Central Role of Neurons in Predicting Severity of Alzheimer’s Disease
Source: PLoS One. 2012 Sep 28;7(9):e45879. doi: 10.1371/journal.pone.0045879 (PMC3461041; doi:10.1371/journal.pone.0045879)
Supplement: Table S1 — Number of genes that were selected for the different feature selection classification models of the various biological processes. (DOC) [file pone.0045879.s003.doc]

**Table S1: Number of genes that were selected for the different feature selection classification models of the various biological processes**.

|  | **Number of genes in feature selection models** | | | | | | | |
| --- | --- | --- | --- | --- | --- | --- | --- | --- |
| **Classification model** | **BR** | **DP** | **RS** | **MCO** | **LO** | **MP** | **IS** | **BA** |
| Neuron- entorhinal cortex | 400 | 200 | 300 | 200 | 50 | 200 | 50 | 100 |
| Neuron-hippocampus | 400 | 200 | 500 | 300 | 100 | 100 | 50 | 200 |
| Whole cortex | 400 | 200 | 200 | 50 | 50 | 400 | 50 | 50 |
| Whole hippocampus | 400 | 300 | 700 | 700 | 150 | 700 | 700 | 200 |
| Astrocyte - cortex | 400 | 400 | 300 | 500 | 50 | 300 | 300 | 300 |

BR: Biological Regulation, DP: Developmental process, RS: Response to Stimulus, MCO: Multi Cellular Organismal process, LO: Localization, MP: Metabolic Process, IS: Immune System process, BA: Biological Adhesion.
