# Supplementary material for: Analyzing Gene Expression from Whole Tissue vs. Different Cell Types Reveals the Central Role of Neurons in Predicting Severity of Alzheimer’s Disease
Source: PLoS One. 2012 Sep 28;7(9):e45879. doi: 10.1371/journal.pone.0045879 (PMC3461041; doi:10.1371/journal.pone.0045879)
Supplement: Table S2 — Mean prediction accuracies obtained by different biological processes classification models by using SVM and decision tree algorithms. (DOC) [file pone.0045879.s004.doc]

**Table S2: Mean prediction accuracies obtained by different biological processes classification models by using SVM and decision tree algorithms.** The models were built using WEKA [1].

| Classification model | SVM | decision tree |
| --- | --- | --- |
| Neuron- entorhinal cortex | 0.92 | 0.8 |
| Whole cortex | 0.86 | 0.74 |
| Astrocyte - cortex | 0.54 | 0.5 |
| Neuron-hippocampus | 0.93 | 0.78 |
| Whole hippocampus | 0.55 | 0.5 |

References

1. Hall M, Frank E, Holmes G, Pfahringer B, Reutemann P, Witten IH. The WEKA data mining software: an update. SIGKDD Explor. Newsl. 11:10–18. (http://www.cs.waikato.ac.nz/ml/weka/)
